# Receiving hemodialysis in Hispanic ethnic dense communities is associated with better adherence and outcomes among young patients: a retrospective analysis of the Dialysis Outcomes and Practice Patterns Study

**DOI:** 10.1186/s12882-023-03297-w

**Published:** 2023-09-05

**Authors:** Ayana K. April-Sanders, Angelo Karaboyas, Milagros Yunes, Keith C. Norris, Mary Dominguez, Ryung S. Kim, Carmen R. Isasi, Ladan Golestaneh

**Affiliations:** 1 Department of Biostatistics and Epidemiology, Rutgers School of Public Health, Piscataway, 683 Hoes Lane West Piscataway, 08854 NJ USA; 2 Arbor Research Collaborative for Health, Ann Arbor, MI USA; 3 Renal Division, Albert Einstein College of Medicine/Montefiore Medical Center, Bronx, NY USA; 4 Division of General Internal Medicine and Nephrology, University of California Los Angeles David Geffen School of Medicine, Los Angeles, CA USA; 5 Department of Epidemiology and Population Health, Albert Einstein College of Medicine, 1300 Morris Park Ave, 10461 Bronx, NY USA

**Keywords:** Hemodialysis, Hispanic ethnic density, Community, Neighborhood integration, Healthy disparities

## Abstract

**Background:**

Hispanic ethnic density (HED) is a marker of better health outcomes among Hispanic patients with chronic disease. It is unclear whether community HED is associated with mortality risk among ethnically diverse patients receiving maintenance hemodialysis.

**Methods:**

A retrospective analysis of patients in the United States cohort of the Dialysis Outcomes and Practice Patterns Study (DOPPS) database (2011–2015) was conducted (n = 4226). DOPPS data was linked to the American Community Survey database by dialysis facility zip code to obtain % Hispanic residents (HED). One way ANOVA and Kruskal Wallis tests were used to estimate the association between tertiles of HED with individual demographic, clinical and adherence characteristics, and facility and community attributes. Multivariable Cox proportional hazards models were used to estimate the mortality hazard ratio (HR) and 95% CIs by tertile of HED, stratified by age; a sandwich estimator was used to account for facility clustering.

**Results:**

Patients dialyzing in facilities located in the highest HED tertile communities were younger (61.4 vs. 64.4 years), more commonly non-White (62.4% vs. 22.1%), had fewer comorbidities, longer dialysis vintage, and were more adherent to dialysis treatment, but had fewer minutes of dialysis prescribed than those in the lowest tertile. Dialyzing in the highest HED tertile was associated with lower hazard of mortality (HR, 0.86; 95% CI, 0.72-1.00), but this association attenuated with the addition of individual race/ethnicity (HR, 0.92; 95% CI, 0.78–1.09). In multivariable age-stratified analyses, those younger than 64 showed a lower hazard for mortality in the highest (vs. lowest) HED tertile (HR, 0.66; 95% CI, 0.49–0.90). Null associations were observed among patients ≥ 64 years.

**Conclusions:**

Treating in communities with greater HED and racial/ethnic integration was associated with lower mortality among younger patients which points to neighborhood context and social cohesion as potential drivers of improved survival outcomes for patients receiving hemodialysis.

**Supplementary Information:**

The online version contains supplementary material available at 10.1186/s12882-023-03297-w.

## Introduction

Members of the Hispanic community carry a large burden of chronic kidney disease (CKD) in the United States. They are at higher risk for CKD progression to end-stage kidney disease (ESKD) than their non-Hispanic White peers, albeit with a great deal of variability depending on the country of origin [1–3]. Living in predominantly Hispanic communities has shown health benefits among all Hispanic patients, which is also referred to as the Hispanic ethnic density (HED) effect or “barrio-advantage” [4]. But the salubrious effect of HED on health outcomes are experienced even in those of non-Hispanic origin [5, 6]. Some have speculated that social integration within a community builds trust and a sense of collective efficacy [7, 8]. There may be less stigmatization and discrimination in communities that are integrated where family and social networks offer social support for adherence behaviors and better mental health. Residence in socially integrated ethnic enclaves may also provide a place where more health-sustaining cultural habits are maintained (e.g., improved nutrition and lower smoking rates) [9]. In some cases, these advantages superseded the influence of material factors such as poverty, difficulty accessing health services, substandard housing and lack of availability and affordability of healthy foods, which serve as barriers to optimal health [10, 11].

Hispanic and Black individuals receiving maintenance dialysis live longer than White individuals[12] even though they have less access to quality pre-kidney failure care and to early transplantation. This phenomenon, however, is dependent on patient age, with young Black patients at disproportionately higher risk of death [12]. Various studies have shown an association between place of residence and mortality among young racial/ethnic minorities receiving maintenance dialysis [13, 14]. Whether treating dialysis patients in HED communities, described by many as an advantage because of higher racial integration, lower stigma attached to ethnic background, and more social cohesion, may supersede any associated socioeconomic and built environmental disadvantage with respect to health outcomes is not well known [8, 9, 15]. We undertook a retrospective cohort study of a national sample of patients receiving maintenance hemodialysis to examine the association between community HED with socio-demographic and clinical attributes, adherence behaviors, and mortality.

## Methods

### Design, setting & participants

**Fig. 1:**
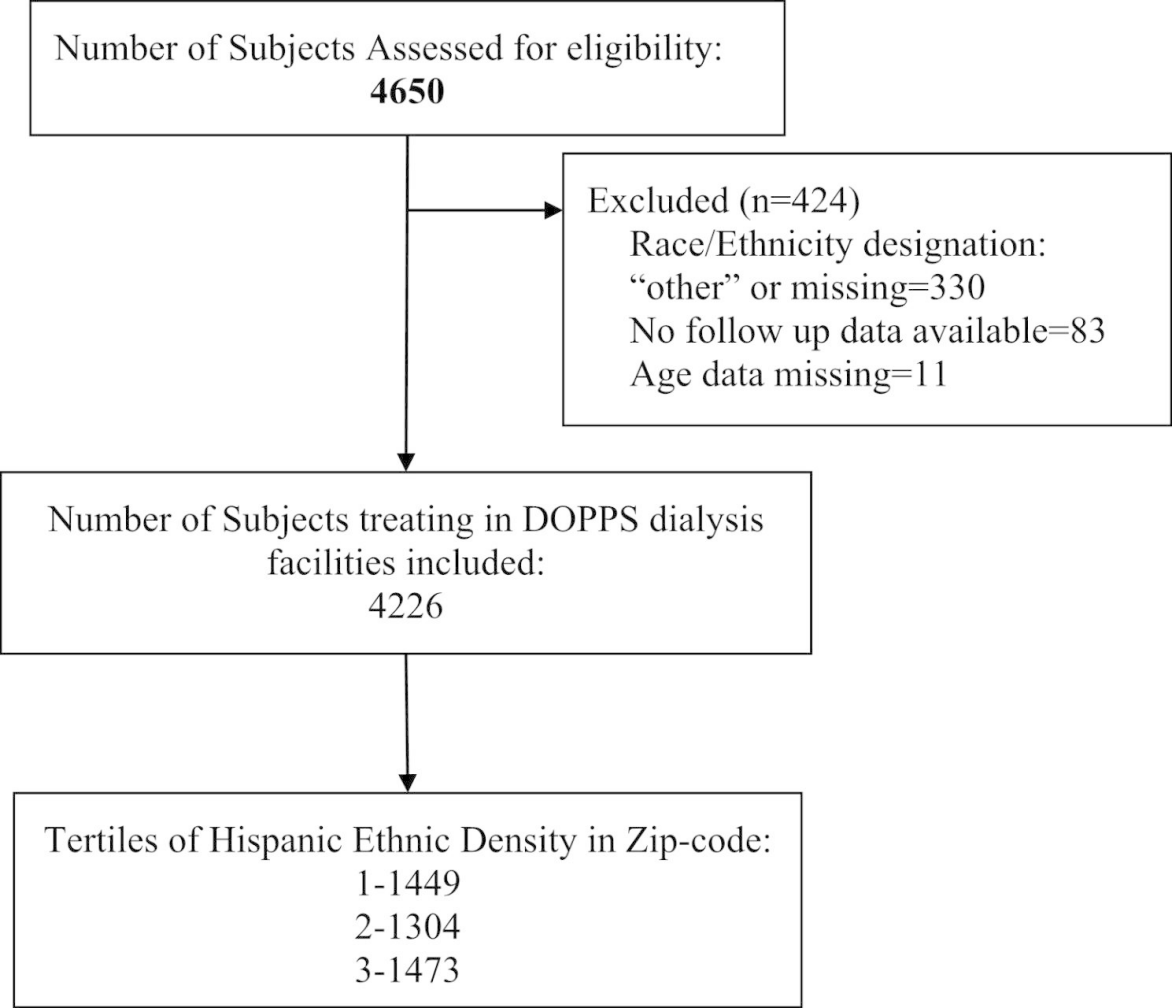
The study cohort

In retrospective cohort analysis, we used patient-level data from the prospectively collected United States subset of the Dialysis Outcomes and Practice Patterns Study (DOPPS) database, during phases 4 and 5 (2010–2015) [16, 17]. The DOPPS randomly selected 20–40 incident and prevalent patients per facility within a random sample of dialysis facilities in the US. Race and ethnicity were obtained from the medical questionnaire completed by study coordinators. After excluding 6 facilities that did not follow up patients, the cohort included 4,650 patients, from which 330 were excluded because of race/ethnicity information designated as “other” or missing; another 11 were excluded because of missing age data. The final cohort was treated in 154 DOPPS dialysis facilities and 127 Zip-codes. (Fig. 1) We obtained institutional review board approval for this study from the Albert Einstein College of Medicine and are in compliance with the Declaration of Helsinki.

### Outcome Variable

We examined death as a time-to-event outcome during follow-up in the DOPPS. Follow up data on death or disenrollment was available through June 2015. All death events were recorded prospectively by the DOPPS study coordinator during follow-up visits.

### Exposure variable

HED was calculated from Census Bureau derived percent Hispanic residents in communities defined by dialysis facility Zip-codes. The dialysis facility data was linked to the American Community Survey (ACS) data gathered over 2011–2015. To account for a significant right skewing of the measures, community HED was classified into tertiles of % Hispanic households in Zip-code based on distribution of the demographic data.

### Other variables

Information on individual level patient demographics, comorbidity history, laboratory values, dialysis treatment parameters and adherence, medication prescriptions, and where available, ethnicity and race, was abstracted from medical records at DOPPS enrollment. Clinical comorbidities, etiology of kidney failure, and other case-mix variables described in Table 1 (e.g., medications, dialysis treatments, laboratory values) were also abstracted from medical records at enrollment. We used a modified Charlson comorbidity score previously reported for analyzing DOPPS data [18, 19]. The DOPPS data included the profit status of and number of patients at each facility at the time of enrollment in the study. Dialysis treatment prescriptions (urea reduction ratio (URR), number of dialysis minutes per week) and adherence (number of missed and shortened dialysis treatments in the previous 30 days) were also included, as were dialysis-related medications and type of access. For community level attributes such as deprivation and urban/rural location, Zip-code level community variables were obtained from the ACS, including mean household size, percent of household income under the poverty line, percent of households with at least one member with a Bachelor’s degree or higher, percent of households with an active internet subscription, percent of households led by a single female, percent of households with Spanish as the primary language, percent of households born in Latin America, and percent of households who immigrated to the US after 2010.

**Table 1:** Baseline Characteristic Differences between Patients Receiving Hemodialysis in Communities Categorized by %Hispanic Ethnic Density (HED), US DOPPS 2010–2015, N = 4226

	HED Tertile 1 % Range: 0–3.7% N = 1449	HED Tertile 2 % Range: 3.8–13.5% N = 1304	HED Tertile 3 % Range: 13.6–91.7% N = 1473	P value
*Individual Clinical/Socio-demographic Variables*				
Age, years (m±SD)	64.4±14.9	63.4±15.0	61.4±15.1	< 0.001
Gender Female	635 (43.8)	598 (45.6)	642 (43.5)	0.5
Race/Ethnicity Asian Hispanic Non-Hispanic Black Non-Hispanic White	4 (0.3) 8 (0.6) 310 (21.4) 1127 (77.8)	129 (9.4) 78 (5.7) 434 (31.7) 728 (53.2)	47 (3.3) 342 (24.3) 490 (34.8) 529 (37.6)	< 0.001
Diabetes	866 (59.7)	782 (59.6)	851 (57.7)	0.5
Heart failure	579 (40.5)	442 (34.9)	605 (43.5)	< 0.001
Hypertension, n = 4090	1266 (88.6)	1088 (85.6)	1156 (83.2)	< 0.001
Psychiatric Disease, n = 4092	357 (24.6)	252 (19.2)	221 (15.0)	< 0.001
Coronary Disease, n = 4092	619 (43.2)	507 (39.9)	501 (36.2)	< 0.001
BMI category, n = 3991 < 23 kg/m^2^ 23–32 kg/m^2^ >=32 kg/m^2^	250 (18.3) 661 (48.3) 456 (33.4)	308 (24.9) 583 (47.1) 346 (28.0)	334 (24.1) 711 (51.3) 342 (24.7)	< 0.001
Charlson Score, n = 3711 < 4 4–6 > 6	234 (18.0) 520 (40.1) 544 (41.9)	213 (18.7) 513 (44.9) 416 (36.4)	251 (19.8) 581 (45.7) 439 (34.5)	0.002
Dialysis Vintage (years) Median (IQR range)]	2.9 (1.3–5.2)	2.9 (1.4–5.3)	3.2 (1.4–5.6)	0.03
Dialysis access, n = 4062 AVF AVG Catheter Other	894 (64.5) 228 (27.8) 257 (18.6) 6 (0.4)	723 (58.0) 281 (22.5) 227 (18.2) 15 (1.2)	848 (59.3) 311 (21.7) 272 (19.0) 0	< 0.001
Insurance, n = 4176 Medicare Medicaid Private VA No Insurance	1156 (81.4) 44 (3.1) 190 (13.4) 28 (2.0) 2 (0.1)	1027 (78.9) 57 (4.4) 187 (14.4) 27 (2.1) 3 (0.2)	1053 (72.4) 104 (7.2) 271 (18.6) 14 (1.0) 13 (0.9)	< 0.001
Substance use in past 12 months, n = 4081	33 (2.3)	23 (1.8)	15 (1.1)	0.04
*Dialysis Related Variables*				
Shortened dialysis within the last 160 days, (n = 3664) 0 1 > 1	1244 (85.7) 198 (13.6) 9 (0.6)	1102 (84.1) 193 (14.7) 12 (1.2)	1317 (89.4) 153 (10.4) 4 (0.3)	< 0.001
Missed dialysis within the last 160 days, (n = 3658) 0 1 > 1	1241 (85.5) 208 (14.3) 2 (0.1)	1100 (83.9) 198 (15.1) 13 (1.0)	1316 (89.2) 154 (10.4) 4 (0.3)	< 0.001
Facility profit status, n = 3437 For-profit	1126 (77.6)	954 (72.8)	1247 (84.5)	< 0.001
Dialysis duration (minutes/week), (m±SD), n = 3977	656.1±96.3	649±101.6	642.7±94.9	< 0.001
URR, m±SD, n = 3897	73.5 (7.4)	73.9 (7.7)	73.5 (7.2)	0.3
Weight loss as a percent of the dry weight in the second treatment of the previous month, [Median (IQR range)], n = 4018	3.0 (1.9-4.0)	3.0 (2.0–4.0)	3.1 (2.1–4.2)	0.01
Number of patients in Facility, [Median (IQR range)], n = 4226	54 (31–66)	65 (47–98)	110 (69–180)	< 0.001
*Community Variables* (m±SD)				
Mean number of family members per household	2.95± 0.20	3.10± 0.26	3.35± 0.21	< 0.001
% household incomes under the poverty line	12.8± 9.0	11.9± 5.5	15.9± 6.1	< 0.001
% of households with Spanish as their primary language	0.83± 1.0	4.1± 3.0	27.8± 16.5	< 0.001
% of households with at least one member with a Bachelors degree or higher	26.7± 13.1	31.9± 18.0	27.2± 11.7	< 0.001
% of households with an active internet subscription	74.5± 10.3	76.9± 8.7	78.0± 7.3	< 0.001
% of households who immigrated after 2010	2.5± 3.8	6.2± 6.1	13.4± 9.5	< 0.001
% of household born in Latin America	0.6± 0.5	2.4± 1.6	14.2± 7.2	< 0.001
% of households led by a single female	12.6± 5.7	12.0± 4.4	15.8± 5.1	< 0.001
% of households that are Black	12.8± 23.0	13.7± 16.0	15.4± 14.8	< 0.001
% of households that are White	80.6± 23.3	65.0± 23.9	44.7± 21.9	< 0.001
% Rurality	32.9± 27.1	22.8± 26.5	9.8± 21.1	< 0.001

### Statistical methods

We used STATA version 15.1 for all analyses. Bivariate comparisons between HED tertiles and variables related to individual socio-demographic and clinical variables, dialysis facility and treatment, and community level ACS attributes were conducted using one-way ANOVA and Kruskal Wallis (when variables were skewed) tests. To estimate the mortality hazard ratio (HR), we used Cox proportional hazards regression models and checked proportional hazards assumptions by examining log-log plots and observed versus expected plots. We used a sandwich estimator to account for facility clustering [20]. We were unable to account for clustering at the Zip-code level because of the requirement that the location of DOPPS affiliated dialysis facilities remain anonymous.

Models were built strategically based on an a priori plan to examine the confounding role of individual, dialysis facility, and community level variables on the primary association (HED tertiles and mortality). Variables were included if they were significantly associated with the exposure in bivariate analyses and/or were shown to be associated with outcomes in previous research. Consistent with previous research,[14] we tested for interaction between the exposure and separately for age and race/ethnicity to elucidate any association differences by these individual characteristics. We observed a significant interaction for age (p = 0.03) but not race/ethnicity (p = 0.2). We then stratified analyses by age at 64 years (sample median). All tests were two-sided.

Three community level variables, percent of households with Spanish as the primary language, percent of households who immigrated to the US after 2010, and percent who were born in Latin America, were left out of multivariate modeling because of concern for collinearity with the exposure variable.

### Missing data

The proportion of missing data was ≤ 5% for most variables apart from the following: number of missed dialysis sessions within the prior 30 days (18%), Charlson score (12.2%) and URR (7.8%). The pattern of missing variables was assumed to be at random. We utilized an iterative imputation approach with chained predictive analytics (10 imputations) to impute missing data [21]. We then performed the regressions with the imputed data and compared the point estimate results to analyses from the non-imputed database in sensitivity analyses. (Supplementary Table 2)

### Exploratory analyses

To gain a better understanding of the intersectionality of age and race/ethnicity with respect to community composition and its relation to mortality, we further stratified the models by race/ethnicity and reported strength and direction of association between community HED and mortality by both age and race. We stratified by Black and White race classifications only as we were unable to stratify by Hispanic ethnicity and Asian race because there were too few individuals in each group.

## Results

### Participants

Among 4226 individuals in the database, the mean ± standard deviation age was 63.1±15.1 years, 56% were male, 56% were White, 10% were Hispanic, 29% were non-Hispanic Black and 4% identified as Asian. For clinical characteristics, 59% had diabetes mellitus, 40% had heart failure, 20% had a psychiatric disorder and 40% were diagnosed with coronary disease. The median BMI was 27.6 and the median Charlson scare was 5 (IQR 4–6).

### Bivariate comparisons I

Patients treated in communities with the highest (vs. lowest) HED tertile were significantly younger (61.4±15.1 vs. 64.4±14.9 years, p < 0.001), had been on dialysis longer (median of 3.2 (IQR, 1.4–5.6) vs. 2.9 years (IQR 1.3–5.2), p = 0.03), had lower Charlson scores and comorbidity (p = 0.002), with fewer that were obese (24.7% vs. 33.4% with BMI > 32, p < 0.001) (Table 1). Prevalence of diabetes did not differ by community type, but diagnosis of psychiatric illness (15.0% vs. 24.6%, p < 0.001) and prevalence of hypertension (83.2% vs. 88.6%, p < 0.001) were lower in HED tertile 3 (vs. tertile 1) communities, while more were diagnosed with heart failure in the highest (vs. lowest) HED tertile (43.5% vs. 40.5%, p < 0.001). Of note, fewer patients in the highest HED communities had a substance use disorder (1.1% in tertile 3 vs. 2.3% in tertile 1, p = 0.04). Patients dialyzing in HED tertile 3 (vs. tertile 1) HED communities were less likely to have Medicare (72.4% vs. 81.4%) and more likely to have Medicaid or no insurance (8.1% vs. 3.2%, p < 0.001).

With respect to dialysis treatments, fewer patients treated in HED tertile 3 shortened or missed dialysis treatments in the 160 days prior to entry into cohort, than patients dialyzing in non-Hispanic communities, p < 0.001 (Table 1). Conversely, patients dialyzing in HED communities were prescribed fewer minutes of dialysis per week (mean 642.7 in tertile 3) as compared to those dialyzing in non-Hispanic communities (mean 656.1 in tertile, p < 0.001); despite this, mean urea reduction ratios were similar between communities, p = 0.3. Dialysis facilities in HED (tertile 3) communities were larger and more commonly for-profit than dialysis facilities in low HED (tertile 1) communities, p < 0.001.

With respect to the socio-economic characteristics of the communities, there were more individuals per household in tertile 3 vs. tertile 1 of HED, with a higher percentage who were below the poverty line, spoke Spanish, were recent immigrants, were born in Latin America and were led by a single female (Table 1, all p < 0.001). Of note, Zip-codes in the highest HED tertile were more integrated communities with a higher percentage of Black residents (14.8% in tertile 3 vs. 12.8% in tertile 1, p < 0.001) and a lower percentage of White residents (44.7% in tertile 3 vs. 80.6% in tertile 1, p < 0.001). Additionally, HED communities were less rural than their low HED counterparts (Table 1). Supplementary Table 1 presents the baseline characteristics stratified by age groups.

### Crude mortality outcomes

A total of 968 (22.9%) deaths occurred over a mean follow-up of 1.35±0.74 years. Crude mortality rates were 0.20/patient years in HED tertile 1, 0.16/patient years in HED tertile 2, and 0.15/patient years in HED tertile 3. Supplemental Table 2 presents the bivariate associations of each variable with mortality.

### Cox Regression Analysis

In the overall sample (Supplemental Table 3), the individual age and sex adjusted hazard ratio of mortality, compared to the reference group of HED tertile 1, was 0.86 (0.72-1.00; p = 0.058) for patients receiving dialysis in HED tertile 3 and 0.82 (95% CI, 0.70–0.97; p = 0.03) for patients receiving dialysis in HED tertile 2. The addition of individual race/ethnicity to the model resulted in attenuation of this association towards the null (HR, 0.93; 95% CI, 0.77–1.07; p = 0.4) in tertile 3 vs. tertile 1 and 0.94 (95% CI, 0.79–1.11; p = 0.7) in tertile 2 vs. tertile 1).

**Fig. 2:**
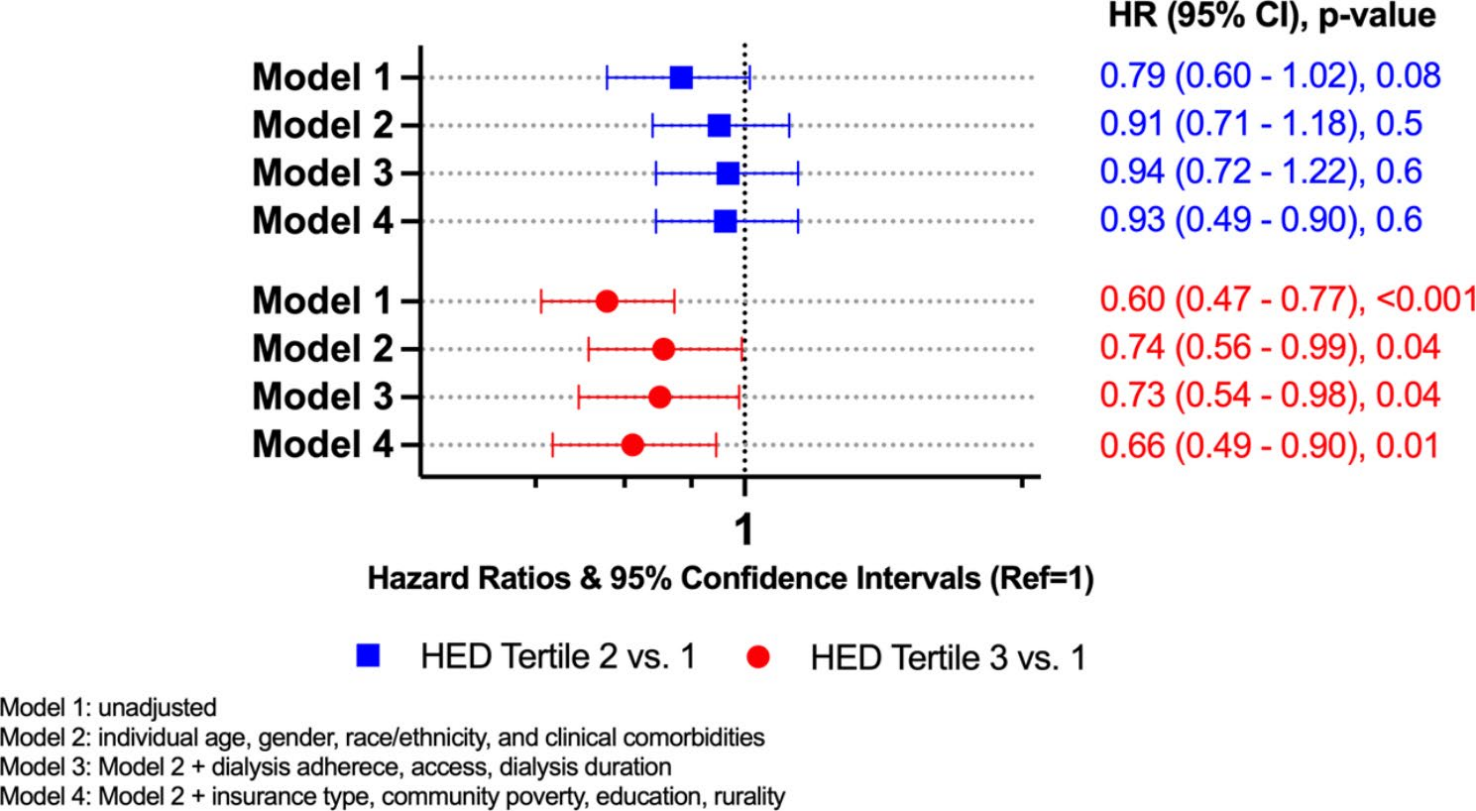
Association between Hispanic ethnic density and hazard of mortality among dialyzing patients < 64 years, DOPPS 2010–2015 (n = 2059)

**Fig. 3:**
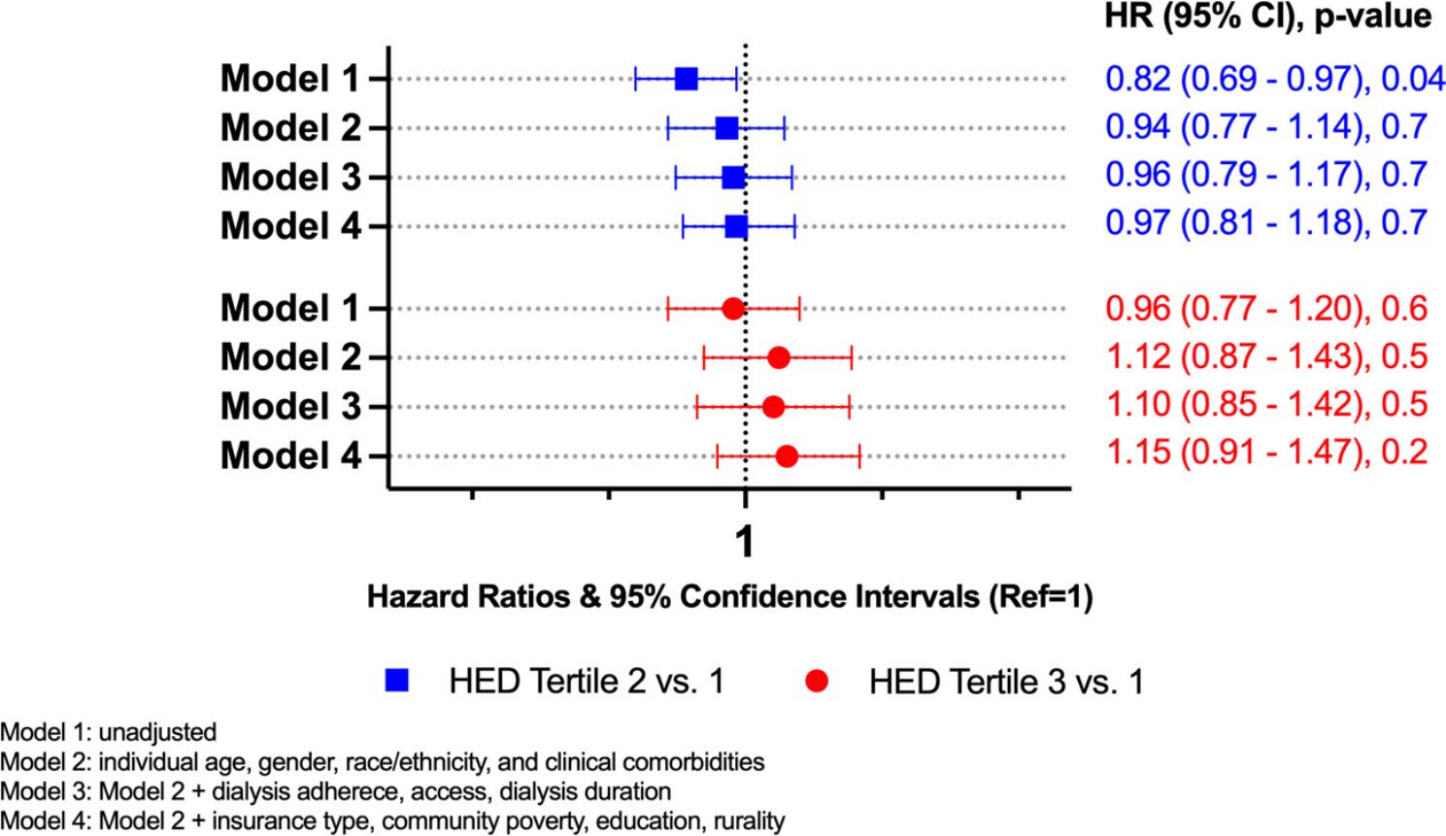
Association between Hispanic ethnic density and hazard of mortality among dialyzing patients ≥ 64 years, DOPPS 2010–2015 (n = 2167)

Among the 51% of patients younger than 64, the unadjusted HR was 0.60 for tertile 3 vs. tertile 1 (95% CI, 0.47–0.77; p < 0.001) (Fig. 2); while for those equal to or older than 64 the unadjusted HR was 0.96 (95% CI, 0.77–1.20; p = 0.6) for tertile 3 vs. tertile 1 (Fig. 3). The association in the younger age group remained robust (HR, 0.66; 95% CI, 0.49–0.90; p = 0.01) after adjustment for individual socio-demographic and clinical characteristics, dialysis related characteristics including adherence, dialysis prescription, type of dialysis access and type of insurance coverage, and community level variables such as rurality, poverty, and education (Fig. 2). By contrast, fully adjusted models showed no difference in the hazard of mortality among the older age group if dialyzing in HED tertile 3 than tertile 1 (HR, 1.15; 95% CI, 0.91–1.47; p = 0.2) (Fig. 3).

In exploratory analyses stratified by age and race/ethnicity, no significant associations were observed. Several trends in hazards were present, however, with imprecise confidence intervals. For example, Black patients, whether younger (HR, 0.69; 95% CI, 0.34-1.00) or older (HR, 0.71; 95% CI, 0.35–1.44), and younger White patients (HR, 0.76; 95% CI 0.51–1.13) had a lower hazard of mortality in the highest HED tertile vs. lowest, while older White patients (HR, 1.28; CI 0.95–1.72) had a higher hazard of mortality; all p-values > 0.05.

## Discussion

The results of this research showed that patients receiving maintenance dialysis in communities with a high HED, while younger and facing higher rates of poverty and less economic opportunity in their communities, had better adherence to dialysis, lower rates of substance use disorder, and lower rates of diagnosed psychiatric illness. Patients younger than 64 years receiving hemodialysis in facilities in high HED had a lower risk for mortality than peers in low HED communities. This association remained robust after adjustment for clinical comorbidity, sociodemographic factors, dialysis quality and adherence, and community level indicators of poverty, education, and rurality.

Communities with higher HED in this study had a higher proportion of households living under the poverty line, lower educational attainment, and other adverse community resource profiles. Patients dialyzing in those communities were also more likely to have higher use of catheters for dialysis access and shorter dialysis duration prescribed, each of which is independently associated with mortality [22]. Furthermore, the higher rate of heart failure in Hispanic communities could be a surrogate for volume overload in a prevalent dialysis population, supporting the notion that fluid removal practices and dialysis prescriptions may not be as aggressive in these communities [23, 24]. Alternatively, higher heart failure prevalence in HED communities may indicate lower access to healthy food items such as those recommended to keep interdialytic weight gain at a low level (low salt diet) and high nutritional value (such as lean meats and plant-based proteins). This phenomenon was supported by higher ultrafiltration volumes observed in those dialyzing in HED tertile 3 than tertile 1. Despite these disadvantages, we did not observe the typical higher mortality risk reported in patients subjected to those adverse community and clinical-level exposures. This finding remained strong for Black patients in our study, similar to other studies showing the health benefits of living in Hispanic areas bridging ethnic divides [25–27]. Factors such as social cohesion related to a higher prevalence of residents with recent immigration status[28] or other socio-cultural factors may have contributed to this phenomenon. It is consistent with the Hispanic health paradox[29, 30] and supported by the lower prevalence of psychiatric disease, substance use, and better adherence observed among those treated in Hispanic (HED tertile 3) compared to non-Hispanic communities (HED tertile 1).

An alternative explanation could also be that racial integration in high HED communities is the driver of lower mortality risk in this cohort. The role of place in health outcomes in those affected by kidney disease has been shown in multiple studies [13, 31–35]. Though neighborhoods in which people live or receive healthcare influence health through material factors such as access, transportation, availability of healthy foods and housing,[12] psychosocial pathways such as social support and social capitol among peers also drive outcomes [15]. For both Black and Hispanic hemodialysis patients, residing in neighborhoods with a concentration of people of similar marginalized race and ethnicity results in a decrease in years lost to heart disease, lower risk of mental illness and cardiovascular death. In our study, the highest tertile of % HED had the highest Black/White integration, with 44.7% White and 15.4% Black residents, than the other tertiles of % HED [6, 27]. Unlike findings from studies that show higher mortality among young patients receiving hemodialysis in communities with lower socio-economic attributes such as social marginalization, poor housing and employment and fewer educational opportunities, our findings provide support for the notion that individuals living in more integrated communities may experience low levels of perceived discrimination and better social integration [9, 36, 37]. Integrated neighborhoods allow diverse racial and ethnic groups to share social spaces and increase social interaction between different groups of people [8]. Through these exchanges, opportunities for social cohesion – exchanging information, building trust, maintaining social support – become more frequent [7]. Factors that have been consistently associated with poorer health and clinical outcomes (e.g., discrimination, implicit bias, referral bias) that predominantly impact minoritized patients and are strongly associated with increased oxidative stress and inflammation and downregulation of immune response[38] can be directly or indirectly mitigated. Integrated neighborhoods are also more likely to hire a diverse network of health-care professionals that can improve patient satisfaction with providers and access to quality care for a diverse patient pool [7].

Analyses performed by the authors using the same cohort showed a higher risk of hospitalization among White and Hispanic patients receiving dialysis in communities with a higher vs. lower percentage of Black residents and a higher risk of hospitalization and mortality among Black men receiving hemodialysis in communities with a high percentage of Black residents as compared to a lower percentage of Black residents [39]. The protective finding of Hispanic ethnic density in this analysis may be due to having a higher proportion of recent immigrant Hispanic residents in addition to the higher integration of different racial/ethnic groups, as mentioned above. Neighborhood immigrant composition is associated with various health-relevant social features linked with communities. Recent immigrants influence social, emotional, language and informational support to Hispanic individuals. For example, social networks and social controls within the neighborhood may collectively reinforce cultural norms on healthy behaviors while discouraging unhealthy activities [40]. Rapid migration of immigrants into multiethnic populations in metropolitan areas can also shift once predominantly White or Black neighborhoods to become more racially integrated areas [6]. Additionally, immigrant enclaves may buffer residents from other ethnic groups from stressful discriminatory exposures or adverse effects of poverty. In general, neighborhoods with high immigrant compositions may have protective effects on health utilization and outcomes [41, 42].

The major strengths of this study include demographic and outcome events using individual patient-level data from a well-established multiracial kidney disease cohort, including Hispanic patients, in the US. We were able to assess mortality outcomes, which is a methodological advantage over past research. This study examined multiple neighborhood-level characteristics by linking ACS data to patient-level data and explored the influence of racially and ethnically integrated neighborhoods on health outcomes, an underexamined approach that could help better understand mechanisms driving racial health inequities [6, 35, 43]. The data also represents real-world practices across many dialysis facilities in a mixture of residential regions.

The current study has several limitations. We recognize that ethnicity is not the same as nativity, and there is important variability in risks and outcomes within the individuals identifying as Hispanic. We included community-level variables related to immigration and citizenship status to better characterize the influence of community attributes common to most Hispanic populations to refine our analyses. We also recognize that dialysis facility Zip codes may not necessarily reflect patients’ residential Zip codes and may have introduced exposure misclassification. While we could not assess concordance between facility and patient residential Zip codes because patients’ addresses were not available in the DOPPS, most patients reside in or close to Zip codes with a dialysis facility [6, 44]. However, referral bias and other socio-economic factors may force patients, especially those from minoritized communities, to dialyze in facilities outside their Zip code. Such locations may have different dialysis facility quality compared to the associated facility with the patient’s residential Zip code [6, 44]. Finally, there were lower rates of diagnosed psychiatric illness reported in patients receiving maintenance dialysis in communities with a high HED, though this may be related to under-reporting of these complications.

## Conclusion

Despite the documented social disadvantage of predominantly Black or Hispanic residential areas, we found that greater residential racial/ethnic integration was associated with lower mortality among younger patients. This association was independent of individual demographic and clinical characteristics, dialysis metrics, and community socio-economic attributes. Neighborhood social capital may allow minoritized patients to navigate, purchase and gain access to necessary dialysis and related health services that otherwise may have been impeded by their limited community-level resources. The current study could not directly examine the influence of neighborhood social resources and connections. However, since neighborhood characteristics are a function of the policies and interventions introduced, future research on neighborhood context and social cohesion could provide insight into the role of the neighborhood social environment and potential strategies that can improve survival outcomes for all patients.

### Electronic supplementary material

Below is the link to the electronic supplementary material.

**Supplementary Table 1**: Age-Stratified (<64 Years, ≥64 Years) Baseline Characteristic Differences between Patients Receiving Hemodialysis in Communities Categorized by %Hispanic Ethnic Density (HED), US DOPPS 2010-2015, N=4226. **Supplementary Table 2**: Bivariate Association Between Each Variable and Mortality Risk. **Supplementary Table 3**: Analysis of Complete Cases Data

## Data Availability

The data that support the findings of this study are available from DOPPS, but restrictions apply to the availability of these data, which were used under license for the current study, and so are not publicly available. Data are, however, available from the authors upon reasonable request and with permission of DOPPS.

## Abbreviations

ACS: American Community Survey
BMI: Body mass index
CI: Confidence interval
DOPPS: Dialysis Outcomes and Practice Patterns Study
ESKD: End-stage kidney disease
HED: Hispanic ethnic density
HR: Hazard ratio
