# Supplementary material for: Receiving hemodialysis in Hispanic ethnic dense communities is associated with better adherence and outcomes among young patients: a retrospective analysis of the Dialysis Outcomes and Practice Patterns Study
Source: BMC Nephrol. 2023 Sep 5;24:263. doi: 10.1186/s12882-023-03297-w (PMC10478353; doi:10.1186/s12882-023-03297-w)
Supplement: Supplementary file 1 — Supplementary Table 1: Age-Stratified (<64 Years, ≥64 Years) Baseline Characteristic Differences between Patients Receiving Hemodialysis in Communities Categorized by %Hispanic Ethnic Density (HED), US DOPPS 2010-2015, N=4226. Supplementary Table 2: Bivariate Association Between Each Variable and Mortality Risk. Supplementary Table 3: Analysis of Complete Cases Data [file 12882_2023_3297_MOESM1_ESM.docx]

## SUPPLEMENTAL MATERIAL PDF

Table of Contents:

Supplementary Table 1: Age-Stratified (<64 Years, ≥64 Years) Baseline Characteristic Differences between Patients Receiving Hemodialysis in Communities Categorized by %Hispanic Ethnic Density (HED), US DOPPS 2010-2015, N=4226

Supplementary Table 2: Bivariate Association Between Each Variable and Mortality Risk

Supplementary Table 3: Analysis of Complete Cases Data

**Supplemental Table 1.** Age-Stratified (<64 Years, ≥64 Years) Baseline Characteristic Differences between Patients Receiving Hemodialysis in Communities Categorized by %Hispanic Ethnic Density (HED), US DOPPS 2010-2015, N=4226

|  | **Age <64 Years** | | |  | **Age ≥64 Years** | | |  |  |
| --- | --- | --- | --- | --- | --- | --- | --- | --- | --- |
|  | **HED**  **Tertile 1**  % Range:  0–3.7%  N= 765 | **HED**  **Tertile 2**  % Range:  3.8–13.5%  N=717 | **HED**  **Tertile 3**  % Range:  13.6–91.7%  N=819 | **p-value** | **HED**  **Tertile 1**  % Range:  0–3.7%  N=846 | **HED**  **Tertile 2**  % Range:  3.8–13.5%  N=718 | **HED**  **Tertile 3**  % Range:  13.6–91.7%  N=690 | **p-value** |  |
| *Individual Clinical/Socio-demographic Variables* | | | | | | | | | |
| Age, years (m±SD) | 50.8±10.6 | 50.8±9.7 | 50.4±10.1 | 0.4 | 75.2±7.3 | 75.0±7.5 | 74.6±7.5 | 0.2 |  |
| Gender  Female | 339 (44.3) | 316 (44.1) | 337 (41.2) | 0.5 | 392 (46.2) | 344 (47.4) | 319 (46.1) | 0.8 |  |
| Race/Ethnicity  Non-Hispanic White  Non-Hispanic Black  Hispanic  Asian | 461 (60.3)  189 (24.7)  3 (0.39)  1 (0.13) | 266 (37.1)  270 (37.7)  51 (7.1)  41 (5.7) | 246 (30.0)  334 (40.8)  197 (24.1)  21 (2.6) | <0.001 | 668 (78.8)  121 (14.3)  5 (0.6)  3 (0.35) | 401 (55.2)  168 (23.1)  25 (3.4)  89 (12.3) | 345 (49.9)  157 (22.7)  149 (21.5)  26 (3.8) | <0.001 |  |
| Diabetes | 485 (63.4) | 423 (59.0) | 430 (52.5) | 0.02 | 522 (61.6) | 465 (64.1) | 442 (63.9) | 0.3 |  |
| Heart failure | 274 (36.2) | 230 (33.2) | 297 (39.2) | 0.03 | 354 (42.6) | 260 (36.9) | 319 (47.8) | <0.001 |  |
| Hypertension, n=4090 | 668 (88.4) | 579 (82.8) | 640 (84.3) | 0.2 | 746 (89.6) | 611 (86.9) | 543 (81.5) | <0.001 |  |
| Psychiatric Disease, n=4092 | 236 (30.9) | 128 (17.9) | 122 (14.9) | <0.001 | 171 (20.2) | 130 (17.9) | 106 (15.3) | 0.03 |  |
| Coronary Disease, n=4092 | 259 (34.1) | 232 (33.3) | 221 (29.3) |  | 430 (51.6) | 315 (44.6) | 292 (43.8) | 0.01 |  |
| BMI category, n=3991  <23 kg/m^2^  23-32 kg/m^2^  >=32 kg/m^2^ | 121 (16.9)  314 (43.7)  283 (39.4) | 152 (22.3)  293 (43.0)  237 (34.8) | 178 (23.1)  365 (47.4)  227 (29.5) | 0.002 | 150 (18.8)  430 (53.9)  218 (27.3) | 184 (26.9)  350 (51.2)  150 (21.9) | 168 (25.8)  362 (55.5)  122 (18.7) | <0.001 |  |
| Charlson Score, n=3711  <4  4-6  >6 | 149 (21.7)  312 (45.4)  227 (33.0) | 138 (21.8)  305 (48.2)  190 (30.0) | 170 (24.6)  321 (46.4)  201 (29.1) | 0.7 | 88 (11.7)  281 (37.4)  383 (50.9) | 91 (14.3)  283 (44.4)  263 (41.3) | 84 (13.9)  278 (45.9)  244 (40.3) | 0.003 |  |
| Dialysis Vintage (years)  Median (IQR range)] | 3.2 (1.3-5.7) | 3.3 (1.6-6.0) | 3.5 (1.6-6.1) | 0.7 | 2.7 (1.3-4.8) | 2.7 (1.3-4.9) | 2.9 (1.4-5.2) | 0.2 |  |
| Dialysis access, n=4062  AVF  AVG  Catheter  Other | 467 (64.2)  121 (16.6)  133 (18.3)  7 (1.0) | 422 (61.9)  128 (18.8)  18.3)  7 (1.0) | 488 (61.2)  158 (19.8)  152 (19.1)  . | 0.1 | 533 (65.8)  129 (15.9)  147 (18.2)  1 (0.1) | 390 (56.2)  176 (25.4)  120 (16.3)  8 (1.2) | 385 (57.6)  159 (23.8)  125 (18.7)  0 | <0.001 |  |
| Insurance, n=4176  Medicare  Medicaid  Private  VA  No Insurance | 573 (76.4)  52 (6.9)  109 (14.5)  14 (1.9)  2 (0.3) | 504 (70.7)  56 (7.9)  135 (18.9)  16 (2.2)  2 (0.3) | 562 (69.4)  84 (10.4)  146 (18.0)  7 (0.9)  11 (1.4) | 0.01 | 716 (86.9)  3 (04)  87 (10.6)  17 (2.1)  1 (0.1) | 615 (85.4)  2 (0.3)  88 (12.2)  14 (1.9)  1 (0.1) | 516 (75.8)  26 (3.8)  130 (19.1)  7 (1.0)  2 (0.3) | <0.001 |  |
| Substance use in past 12 months, n=4081 | 37 (4.9) | 19 (2.8) | 11 (1.5) | 0.001 | 3 (0.4) | 5 (0.7) | 4 (0.6) | 0.2 |  |
| *Dialysis Related Variables* | | | | | | | | | |
| Shortened dialysis within the last 160 days, (n=3664)  0  1  >1 | 632 (82.7)  117 (15.3)  15 (1.96) | 628 (87.6)  82 (11.4)  7 (0.98) | 726 (88.8)  88 (10.8)  4 (0.49) | 0.1 | 702 (82.9)  143 (16.9)  2 (0.2) | 601 (82.9)  115 (15.9)  9 (1.24) | 616 (89.0)  76 (11.0)  0 | 0.01 |  |
| Missed dialysis within the last 160 days, (n=3658)  0  1  >1 | 631 (82.5)  123 (16.1)  11 (1.4) | 627 (87.5)  83 (11.6)  7 (1.0) | 725 (88.5)  90 (11.0)  4 (0.5) | 0.1 | 701 (82.9)  143 (16.9)  2 (0.2) | 600 (82.6)  120 (16.5)  6 (0.8) | 616 (89.0)  74 (10.7)  2 (0.3) | 0.4 |  |
| Facility profit status, n=3437  For-profit | 563 (73.6) | 553 (77.1) | 707 (86.3) | <0.001 | 625 (73.7) | 525 (72.3) | 558 (80.6) | <0.001 |  |
| Dialysis duration (minutes/week),  (m±SD), n=3977 | 669.6±97.0 | 670.7±101.0 | 652.9±100.4 | <0.001 | 640.0±91.5 | 632.4±97.0 | 629.0±85.2 | 0.02 |  |
| URR, m±SD, n=3897 | 72.7±7.7 | 72.9±7.9 | 72.5±7.9 |  | 74.3±6.9 | 74.7±7.2 | 74.5±6.4 |  |  |
| Weight loss as a percent of the dry weight in the second treatment of the previous month, [Median (IQR range)], n=4018 | 3.2 (2.2-4.3) | 3.3 (2.1-4.3) | 3.3 (2.3-4.4) | 0.1 | 2.8 (1.8-3.8) | 2.9 (1.9-3.9) | 3.0 (2.0-3.9) | 0.1 |  |
| Number of patients in Facility,  [Median (IQR range)], n=4226 | 49 (31-65) | 74 (49-103) | 110 (69-180) | <0.001 | 56 (31-66) | 65 (47-103) | 110 (68-158) | <0.001 |  |
| *Community Variables* (m±SD) | | | | | | | | | |
| Mean number of family members per household | 3.1±0.5 | 3.1±0.3 | 3.4±0.2 | <0.001 | 3.0±0.4 | 3.1±0.3 | 3.3±0.2 | <0.001 |  |
| % household incomes under the poverty line | 15.1±10.8 | 12.4±6.2 | 16.4±6.3 | <0.001 | 12.9±8.6 | 12.0±5.4 | 15.1±6.0 | <0.001 |  |
| % of households with Spanish as their primary language | 0.9±1.1 | 3.8±2.9 | 27.9±16.6 | <0.001 | 0.8±0.9 | 4.1±3.0 | 27.6±16.4 | <0.001 |  |
| % of households with at least one member with a Bachelors degree or higher | 24.7±12.3 | 33.1±19.2 | 27.1±12.0 | <0.001 | 26.8±13.4 | 29.9±15.6 | 28.0±12.4 | <0.001 |  |
| % of households with an active internet subscription | 71.2±12.2 | 76.3±9.6 | 77.6±7.8 | <0.001 | 74.7±10.3 | 76.7±8.8 | 78.7±6.8 | <0.001 |  |
| % of households who immigrated after 2010 | 2.2±3.6 | 7.0±6.5 | 13.4±9.5 | <0.001 | 2.4±3.8 | 6.4±6.4 | 13.8±10.0 | <0.001 |  |
| % of household born in Latin America | 2.7±2.8 | 11.6±10.4 | 23.1±10.8 | <0.001 | 3.1±3.1 | 11.5±10.7 | 23.1±11.0 | <0.001 |  |
| % of households led by a single female | 14.9±7.9 | 12.2±4.8 | 16.2±5.3 | <0.001 | 13.0±6.6 | 12.3±3.9 | 15.2±4.9 | <0.001 |  |
| % of households that are Black | 14.4±23.3 | 14.3±16.5 | 16.9±15.8 | 0.2 | 10.4±21.1 | 11.1±14.6 | 13.3±13.2 | 0.01 |  |
| % of households that are White | 72.6±29.5 | 59.2±26.1 | 42.7±22.7 | <0.001 | 79.9±25.1 | 63.0±27.3 | 47.3±20.7 | <0.001 |  |
| % Rurality | 37.5±29.3 | 22.8±28.4 | 10.7±22.2 | <0.001 | 33.3±28.2 | 22.7±27.1 | 8.5±19.6 | <0.001 |  |

**Supplementary Table 2:** Bivariate Association Between Each Variable and Mortality Risk

| N=430 | HR for mortality  (95% CI) | p-value |
| --- | --- | --- |
| ***Demographic*** |  |  |
| Age (per year) | 1.04 (1.03-1.04) | <0.001 |
| Gender (male vs female) | 1.15 (1.02-1.03) | 0.03 |
| Race/Ethnicity  Non-Hispanic White  Non-Hispanic Black  Hispanic  Asian | 1  0.45 (0.36-0.57)  0.54 (0.42-0.70)  0.65 (0.53-0.81) | <0.001  <0.001  0.01 |
| BMI kg/m^2^ (N=3981) | 0.97 (0.96-0.98) | <0.001 |
| *Clinical Comorbidity* |  |  |
| Coronary disease (yes vs no) (N=4081) | 1.63 (1.31-1.89) | <0.001 |
| Diabetes Mellitus (yes vs no) | 1.45 (1.26-1.67) | <0.001 |
| Heart Failure (yes vs no) (N=4076) | 1.73 (1.51-1.98) | <0.001 |
| Hypertension (yes vs no) (N=4079) | 0.94 (0.77-1.15) | 0.6 |
| Psychiatric Diagnosis (yes vs no) | 1.28 (1.09-1.51) | 0.003 |
| Charlson Score (N=3702) | 1.22(1.17-1.26) | <0.001 |
| ***Dialysis related*** |  |  |
| URR (per 10 units) (N=3977) | 1.04 (0.95-1.13) | 0.4 |
| Dialysis Duration (per 10 more minutes prescribed per week) (N=3977) | 0.99 (0.96-0.99) | 0.007 |
| AVF Access (N=4051)  AVG  Catheter  Other | 1  1.06 (0.90-1.24)  1.43 (1.25-1.64)  1.01 (0.62-1.67) | 0.5  <0.001  0.96 |
| Missed dialysis >=1 in 160 days of enrollment (N=3658) | 0.97 (0.80-1.18) | 0.8 |
| Shortened dialysis >=1 in 160 days of enrollment (N=3664) | 1.06 (0.88-1.28) | 0.5 |
| Non-profit (vs for-profit) facility status | 1.13 (0.95-1.35) | 0.2 |
| Facility census (per10 units) | 0.98 (0.96-0.99) | <0.001 |
| ***Community related*** |  |  |
| % of resident household incomes under the poverty line, per decile | 0.99 (0.91-1.07) | 0.76 |
| % with at least one member of household with a Bachelor’s degree or higher, per decile | 0.94 (0.90-0.99) | 0.01 |
| % of households with Spanish as primary language, per decile | 0.99 (0.99-1.00) | 0.19 |
| % of households who were immigrants after 2010, per decile | 0.93 (0.86-0.99) | 0.04 |
| % of households led by a single female, per decile | 0.90 (0.81-1.00) | 0.05 |
| % rurality, per decile | 1.03 (1.01-1.06) | 0.004 |

*Scale – per 1 unit increase, unless otherwise stated

**Supplemental Table 3:** Analysis of Complete Cases Data

| N=4226^†^ | **Model 1**:  Unadjusted | **Model 2**:  Adjusted for individual  Age, Gender, race/ethnicity, and clinical comorbidity | **Model 3**:  Model 2+ adjustment for dialysis adherence, access, dialysis duration | **Model 4:**  Model 2+ adjustment for insurance type, community poverty, education, rurality |
| --- | --- | --- | --- | --- |
| All  Tertile 1  Tertile 2  Tertile 3 | N=4226  1  0.82 (0.70-0.96)^*^  0.86 (0.72-1.02) | N=4060  1  0.94 (0.79-1.11)  0.93 (0.77-1.07) | N=3743  1  0.95 (0.78-1.16)  0.88 (0.69-1.13) | N=4000  1  0.97 (0.82-1.14)  0.92 (0.77-1.10) |
| *Age <64 (n=2059)*  % HED  Tertile 1  Tertile 2  Tertile 3 | N=2059  1  0.79 (0.60-1.02)  0.60 (0.47-0.77)^***^ | N=1960  1  0.93 (0.72-1.21)  0.71 (0.54-0.96)^*^ | N=1805  1  0.89 (0.67-1.19)  0.67 (0.50-0.91)^**^ | N=1937  1  0.92 (0.71-1.21)  0.63 (0.37-0.84)^***^ |
| *Age >=64 (n=2167)*  % HED  Tertile 1  Tertile 2  Tertile 3 | N=2167  1  0.82 (0.69-0.97)^**^  0.96 (0.77-1.20) | N=2100  1  0.96 (0.78-1.18)  1.05 (0.84-1.31) | N=1939  1  0.87 (0.71-1.07)  0.99 (0.78-1.26) | N=2063  1  0.97 (0.80-1.18)  1.06 (0.85-1.33) |

^†^Total sample includes 4226 patients. As model adjustments evolve, the number of patients with complete data change. Each model sample size is included.

^*^p<0.05

^**^p<0.01

^***^p<0.001
